# 4-Iodo­benzohydrazide

**DOI:** 10.1107/S1600536808033898

**Published:** 2008-10-25

**Authors:** Rifat Ara Jamal, Uzma Ashiq, Muhammad Nadeem Arshad, Zahida Tasneem Maqsood, Islam Ullah Khan

**Affiliations:** a Department of Chemical Engineering, NED University of Engineering and Technology, Karachi 75270, Pakistan; b Department of Mathematics and Basic Sciences, NED University of Engineering and Technology, Karachi 75270, Pakistan; c Department of Chemistry, Government College University, Lahore, Pakistan; d Department of Chemistry, University of Karachi, Karachi 75270, Pakistan

## Abstract

In the structure of the title compound, C_7_H_7_IN_2_O, the hydrazide group is inclined at 13.3 (3)° with respect to the benzene ring. The structure is stabilized by inter­molecular N—H⋯N and N—H⋯O hydrogen bonds involving the hydrazide group, resulting in six- and ten-membered rings with *R*_2_^2^(6) and *R*_2_^2^(10) graph-set notations, respectively.

## Related literature

For related structures, see: Kallel *et al.* (1992 ▶); Saraogi *et al.* (2002 ▶); Ashiq, Jamal *et al.* (2008 ▶). For related literature, see: Ara *et al.* (2007 ▶); Ashiq, Ara *et al.* (2008 ▶); Bernstein *et al.* (1994 ▶).
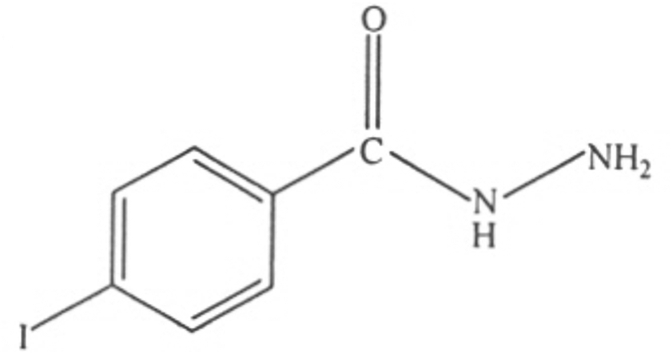


## Experimental

### 

#### Crystal data

C_7_H_7_IN_2_O
*M* *_r_* = 262.05
Monoclinic, 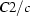

*a* = 28.4394 (18) Å
*b* = 4.4514 (3) Å
*c* = 13.3216 (9) Å
β = 94.292 (2)°
*V* = 1681.72 (19) Å^3^
*Z* = 8
Mo *K*α radiation
μ = 3.76 mm^−1^
*T* = 296 (2) K
0.12 × 0.08 × 0.06 mm

#### Data collection

Bruker KappaAPEXII CCD diffractometer
Absorption correction: multi-scan (*SADABS*; Bruker, 2005 ▶) *T*_min_ = 0.581, *T*_max_ = 0.806
9236 measured reflections
2069 independent reflections
1645 reflections with *I* > 2σ(*I*)
*R*_int_ = 0.030

#### Refinement

*R*[*F* ^2^ > 2σ(*F* ^2^)] = 0.029
*wR*(*F* ^2^) = 0.106
*S* = 1.05
2069 reflections
109 parameters
3 restraints
H atoms treated by a mixture of independent and constrained refinement
Δρ_max_ = 0.55 e Å^−3^
Δρ_min_ = −1.33 e Å^−3^

### 

Data collection: *APEX2* (Bruker, 2007 ▶); cell refinement: *APEX2*; data reduction: *SAINT* (Bruker, 2007 ▶); program(s) used to solve structure: *SHELXS97* (Sheldrick, 2008 ▶); program(s) used to refine structure: *SHELXL97* (Sheldrick, 2008 ▶); molecular graphics: *ORTEP-3 for Windows* (Farrugia, 1997 ▶); software used to prepare material for publication: *SHELXL97*.

## Supplementary Material

Crystal structure: contains datablocks I, global. DOI: 10.1107/S1600536808033898/pv2109sup1.cif

Structure factors: contains datablocks I. DOI: 10.1107/S1600536808033898/pv2109Isup2.hkl

Additional supplementary materials:  crystallographic information; 3D view; checkCIF report

## Figures and Tables

**Table 1:** Hydrogen-bond geometry (Å, °)

*D*—H⋯*A*	*D*—H	H⋯*A*	*D*⋯*A*	*D*—H⋯*A*
N1—H1*A*⋯N2^i^	0.857 (10)	2.19 (3)	2.964 (5)	151 (5)
N2—H2*A*⋯O1^ii^	0.862 (10)	2.240 (14)	3.094 (5)	170 (5)
C3—H3⋯O1^iii^	0.93	2.56	3.257 (5)	132

Symmetry codes: (i) 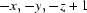; (ii) 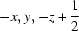; (iii) 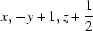.

**Fig. 1.**
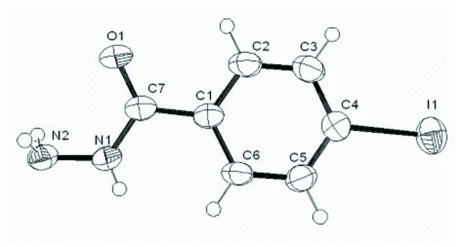
ORTEP plot of the title compound with the ellipsoids drawn at the 50% probability level.

**Fig. 2.**
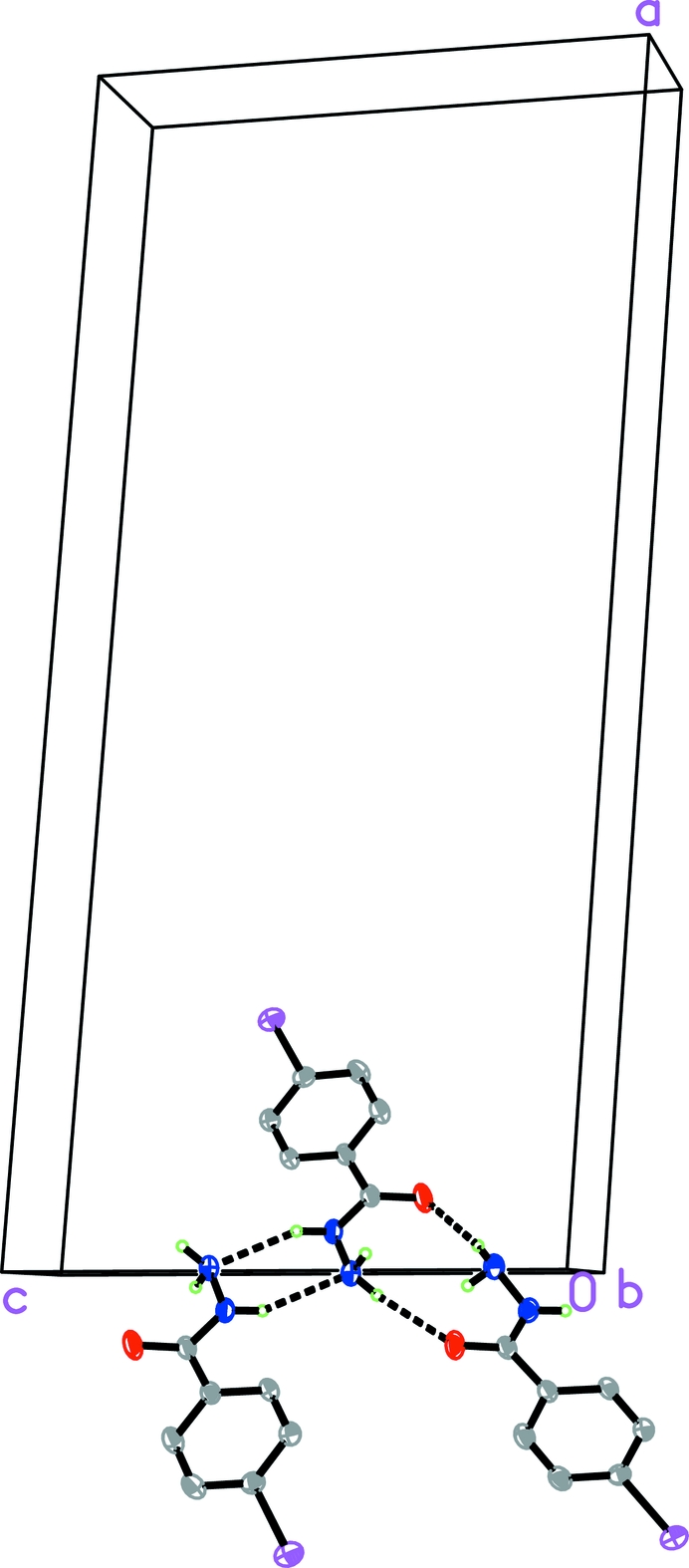
The hydrogen bonding patterns of (I) represented by dashed lines in the unit cell; H-atoms not involved in H-bonds have been excluded.

## supplementary crystallographic information

### Comment

The title compound and its oxovanadium(IV) complex were investigated for their α-glucosidase inhibitory and urease activities. Free hydrazide ligand was found to be inactive, whereas its oxovanadium(IV) complex was found to be a potent inhibitor of α-glucosidase (Ashiq, Ara *et al.*, 2008) and urease (Ara *et al.*, 2007). Continuing our studies on the enzyme inhibition behavior of the title compound, (I), and to investigate the change in its activity due to complexation with vanadium center, we have synthesized (I) and report its crystal structure in this paper. The structures of benzhydrazide (Kallel *et al.*, 1992), *para*-chloro (Saraogi *et al.*, 2002) and *para*-bromo (Ashiq, Jamal *et al.*, 2008) analogues of (I) have already been reported.

The molecule of the title compound (Fig. 1) is far from planar as is evident from the dihedral angle of 13.3 (3)° between the mean-planes of the phenyl ring (C1-C6) and the hydrazide moiety (N1/N2/O1/C7). The bond distances and bond angles in (I) are similar to the corersponding distances and angles reported in the structures quoted above. The molecules of (I) are involved in two types of hydrogen bonds involving hydrazide moiety. On  one hand, the molecules lying about inversion centers form six membered rings *via* N1—H1A···N2^i^ hydrogen bonding. On the other hand, the molecules related by c-glide form ten membered rings *via* N2—H2A···O1^ii^; detail of the hydrogen bonding have been presented in Table 1 and depicted in Fig. 2. The six and ten membered rings represent R_2_^2^(6) and R_2_^2^(10) graph set patterns, respectively (Bernstein *et al.*, 1994).

### Experimental

All reagent-grade chemicals were obtained from Aldrich and Sigma Chemical companies and were used without further purification. To a solution of ethyl-4-iodobenzoate (5.5 g, 20 mmol) in 75 ml ethanol, hydrazine hydrate (5.0 ml, 100 mmol) was added. The mixture was refluxed for 5 h and a solid was obtained upon removal of the solvent by rotary evaporation. The resulting solid was washed with hexane to afford 4-iodobenzohydrazide (yield 84%).

### Refinement

H-atoms bonded to N-atoms were located from a difference map and were included in the refinement at those positions (using DFIX command with N—H = 0.86 (1) Å) while the aryl H-atoms were positioned geometrically in a riding mode, with C—H = 0.93 Å; for all H-atoms, U_iso_ = 1.2 times U_eq_ of the parent atoms.

### Crystal data

**Table:**

C_7_H_7_IN_2_O	*F*(000) = 992
*M**_r_* = 262.05	*D*_x_ = 2.072 Mg m^−^^3^
Monoclinic, *C*2/*c*	Mo *K*α radiation, λ = 0.71073 Å
Hall symbol: -C 2yc	Cell parameters from 3495 reflections
*a* = 28.4394 (18) Å	θ = 1.4–28.3°
*b* = 4.4514 (3) Å	µ = 3.76 mm^−^^1^
*c* = 13.3216 (9) Å	*T* = 296 K
β = 94.292 (2)°	Needle, colorless
*V* = 1681.72 (19) Å^3^	0.12 × 0.08 × 0.06 mm
*Z* = 8	

### Data collection

**Table:**

Bruker KappaAPEXII CCD diffractometer	2069 independent reflections
Radiation source: fine-focus sealed tube	1645 reflections with *I* > 2σ(*I*)
graphite	*R*_int_ = 0.030
ω scans	θ_max_ = 28.3°, θ_min_ = 1.4°
Absorption correction: multi-scan (SADABS; Bruker, 2005)	*h* = −37→37
*T*_min_ = 0.581, *T*_max_ = 0.806	*k* = −5→5
9236 measured reflections	*l* = −17→17

### Refinement

**Table:**

Refinement on *F*^2^	Primary atom site location: structure-invariant direct methods
Least-squares matrix: full	Secondary atom site location: difference Fourier map
*R*[*F*^2^ > 2σ(*F*^2^)] = 0.029	Hydrogen site location: inferred from neighbouring sites
*wR*(*F*^2^) = 0.106	H atoms treated by a mixture of independent and constrained refinement
*S* = 1.05	*w* = 1/[σ^2^(*F*_o_^2^) + (0.0565*P*)^2^ + 5.77*P*] where *P* = (*F*_o_^2^ + 2*F*_c_^2^)/3
2069 reflections	(Δ/σ)_max_ = 0.001
109 parameters	Δρ_max_ = 0.55 e Å^−^^3^
3 restraints	Δρ_min_ = −1.32 e Å^−^^3^

### Special details

**Table:**

Geometry. All esds (except the esd in the dihedral angle between two l.s. planes) are estimated using the full covariance matrix. The cell esds are taken into account individually in the estimation of esds in distances, angles and torsion angles; correlations between esds in cell parameters are only used when they are defined by crystal symmetry. An approximate (isotropic) treatment of cell esds is used for estimating esds involving l.s. planes.
Refinement. Refinement of *F*^2^ against ALL reflections. The weighted *R*-factor *wR* and goodness of fit *S* are based on *F*^2^, conventional *R*-factors *R* are based on *F*, with *F* set to zero for negative *F*^2^. The threshold expression of *F*^2^ > σ(*F*^2^) is used only for calculating *R*-factors(gt) *etc*. and is not relevant to the choice of reflections for refinement. *R*-factors based on *F*^2^ are statistically about twice as large as those based on *F*, and *R*- factors based on ALL data will be even larger.

### Fractional atomic coordinates and isotropic or equivalent isotropic displacement parameters (Å^2^)

**Table:**

	*x*	*y*	*z*	*U*_iso_*/*U*_eq_	
I1	0.215538 (11)	1.09655 (7)	0.64904 (2)	0.05339 (15)	
O1	0.05919 (14)	0.3811 (8)	0.2873 (2)	0.0582 (10)	
N1	0.03174 (13)	0.1851 (9)	0.4262 (2)	0.0404 (8)	
H1A	0.0321 (18)	0.170 (11)	0.4905 (9)	0.049*	
N2	−0.00239 (14)	0.0017 (10)	0.3743 (2)	0.0408 (8)	
H2A	−0.0195 (16)	0.119 (9)	0.335 (3)	0.049*	
H2B	0.0127 (17)	−0.126 (9)	0.341 (3)	0.049*	
C1	0.09627 (14)	0.5363 (10)	0.4445 (3)	0.0358 (8)	
C2	0.09382 (15)	0.5693 (10)	0.5484 (3)	0.0403 (9)	
H2	0.0695	0.4777	0.5799	0.048*	
C3	0.12697 (15)	0.7356 (11)	0.6046 (3)	0.0441 (10)	
H3	0.1244	0.7611	0.6733	0.053*	
C4	0.16380 (15)	0.8638 (9)	0.5594 (3)	0.0397 (9)	
C5	0.16696 (17)	0.8370 (11)	0.4566 (3)	0.0489 (11)	
H5	0.1918	0.9256	0.4260	0.059*	
C6	0.13267 (18)	0.6768 (12)	0.3998 (3)	0.0489 (11)	
H6	0.1342	0.6637	0.3305	0.059*	
C7	0.06129 (16)	0.3608 (9)	0.3801 (3)	0.0375 (9)	

### Atomic displacement parameters (Å^2^)

**Table:**

	*U*^11^	*U*^22^	*U*^33^	*U*^12^	*U*^13^	*U*^23^
I1	0.0461 (2)	0.0498 (2)	0.0626 (2)	−0.00097 (13)	−0.00736 (14)	0.00125 (13)
O1	0.069 (2)	0.080 (3)	0.0273 (13)	−0.0221 (19)	0.0124 (14)	−0.0003 (14)
N1	0.0469 (19)	0.0465 (19)	0.0277 (14)	−0.0046 (17)	0.0010 (13)	0.0067 (14)
N2	0.050 (2)	0.0425 (19)	0.0302 (15)	0.0003 (17)	0.0039 (14)	0.0051 (15)
C1	0.0384 (19)	0.038 (2)	0.0317 (17)	0.0053 (17)	0.0080 (14)	0.0015 (16)
C2	0.039 (2)	0.051 (3)	0.0317 (17)	−0.0001 (18)	0.0109 (15)	0.0041 (17)
C3	0.043 (2)	0.054 (3)	0.0352 (18)	0.001 (2)	0.0049 (16)	0.0000 (19)
C4	0.036 (2)	0.039 (2)	0.044 (2)	0.0035 (16)	−0.0004 (16)	0.0018 (17)
C5	0.048 (2)	0.053 (3)	0.047 (2)	−0.008 (2)	0.0168 (19)	0.002 (2)
C6	0.055 (3)	0.056 (3)	0.038 (2)	−0.004 (2)	0.0156 (19)	0.001 (2)
C7	0.042 (2)	0.041 (2)	0.0309 (17)	0.0060 (17)	0.0088 (15)	0.0037 (15)

### Geometric parameters (Å, °)

**Table:**

I1—C4	2.098 (4)	C1—C7	1.486 (6)
O1—C7	1.237 (5)	C2—C3	1.375 (6)
N1—C7	1.332 (5)	C2—H2	0.9300
N1—N2	1.410 (6)	C3—C4	1.371 (6)
N1—H1A	0.857 (10)	C3—H3	0.9300
N2—H2A	0.862 (10)	C4—C5	1.384 (6)
N2—H2B	0.860 (10)	C5—C6	1.386 (7)
C1—C6	1.382 (6)	C5—H5	0.9300
C1—C2	1.398 (5)	C6—H6	0.9300
			
C7—N1—N2	123.3 (3)	C2—C3—H3	120.0
C7—N1—H1A	123 (3)	C3—C4—C5	120.5 (4)
N2—N1—H1A	114 (3)	C3—C4—I1	118.8 (3)
N1—N2—H2A	106 (3)	C5—C4—I1	120.7 (3)
N1—N2—H2B	107 (4)	C4—C5—C6	119.3 (4)
H2A—N2—H2B	112 (5)	C4—C5—H5	120.4
C6—C1—C2	118.3 (4)	C6—C5—H5	120.4
C6—C1—C7	118.6 (3)	C1—C6—C5	121.1 (4)
C2—C1—C7	123.1 (4)	C1—C6—H6	119.5
C3—C2—C1	120.8 (4)	C5—C6—H6	119.5
C3—C2—H2	119.6	O1—C7—N1	121.3 (4)
C1—C2—H2	119.6	O1—C7—C1	121.3 (4)
C4—C3—C2	120.0 (4)	N1—C7—C1	117.4 (3)
C4—C3—H3	120.0		
			
C6—C1—C2—C3	−0.5 (7)	C7—C1—C6—C5	−178.3 (4)
C7—C1—C2—C3	−179.6 (4)	C4—C5—C6—C1	−1.9 (8)
C1—C2—C3—C4	−2.0 (7)	N2—N1—C7—O1	2.7 (7)
C2—C3—C4—C5	2.5 (7)	N2—N1—C7—C1	−178.5 (4)
C2—C3—C4—I1	−176.8 (3)	C6—C1—C7—O1	−12.8 (6)
C3—C4—C5—C6	−0.6 (7)	C2—C1—C7—O1	166.3 (4)
I1—C4—C5—C6	178.8 (4)	C6—C1—C7—N1	168.4 (4)
C2—C1—C6—C5	2.4 (7)	C2—C1—C7—N1	−12.4 (6)

### Hydrogen-bond geometry (Å, °)

**Table:**

*D*—H···*A*	*D*—H	H···*A*	*D*···*A*	*D*—H···*A*
N1—H1A···N2^i^	0.86 (1)	2.19 (3)	2.964 (5)	151 (5)
N2—H2A···O1^ii^	0.86 (1)	2.24 (1)	3.094 (5)	170 (5)
C3—H3···O1^iii^	0.93	2.56	3.257 (5)	132

Symmetry codes: (i) −*x*, −*y*, −*z*+1; (ii) −*x*, *y*, −*z*+1/2; (iii) *x*, −*y*+1, *z*+1/2.
